# Antimicrobial Effects of a Lipophilic Fraction and Kaurenoic Acid Isolated from the Root Bark Extracts of *Annona senegalensis*

**DOI:** 10.1155/2012/831327

**Published:** 2012-05-24

**Authors:** Theophine Chinwuba Okoye, Peter Achunike Akah, Charles Ogbonnaya Okoli, Adaobi Chioma Ezike, Edwin Ogechukwu Omeje, Uchenna Estella Odoh

**Affiliations:** ^1^Department of Pharmacology and Toxicology, Faculty of Pharmaceutical Sciences, University of Nigeria, Enugu State, Nsukka 410001, Nigeria; ^2^Department of Pharmaceutical and Medicinal Chemistry, Faculty of Pharmaceutical Sciences, University of Nigeria, Enugu State, Nsukka 410001, Nigeria; ^3^Department of Pharmacognosy and Environmental Medicine, Faculty of Pharmaceutical Sciences, University of Nigeria, Enugu State, Nsukka 410001, Nigeria

## Abstract

Root bark preparation of *Annona senegalensis* Pers. (Annonaceae) is used in Nigerian ethnomedicine for treatment of infectious diseases. Extraction of the *A. senegalensis* powdered root bark with methanol-methylene chloride (1 : 1) mixture yielded the methanol-methylene extract (MME) which was fractionated to obtain the ethyl acetate fraction (EF). The EF on further fractionation gave two active subfractions, F1 and F2. The F1 yielded a lipophilic oily liquid while F2 on purification, precipitated white crystalline compound, AS2. F1 was analyzed using GC-MS, while AS2 was characterized by proton NMR and X-ray crystallography. Antibacterial and antifungal studies were performed using agar-well-diffusion method with 0.5 McFarland standard and MICs calculated. GC-MS gave 6 major constituents: kaur-16-en-19-oic acid; 1-dodecanol; 1-naphthalenemethanol; 6,6-dimethyl-bicyclo[3.1.1]hept-2-ene-2-ethanol; 3,3-dimethyl-2-(3-methylbuta-1,3-dienyl)cyclohexane-1-methanol; 3-hydroxyandrostan-17-carboxylic acid. AS2 was found to be kaur-16-en-19-oic acid. The MICs of EF, F1, and AS2 against *B. subtilis* were 180, 60, and 30 **μ**g/mL, respectively. AS2 exhibited activity against *S. aureus* with an MIC of 150 **μ**g/mL, while F1 was active against *P. aeruginosa* with an MIC of 40 **μ**g/mL. However, the extracts and AS2 exhibited no effects against *Candida albicans* and *Aspergillus niger*. Therefore, kaurenoic acid and the lipophilic fraction from *A. senegalensis* root bark exhibited potent antibacterial activity.

## 1. Introduction

The increase in the incidence of new and reemerging infectious diseases caused by organisms with high resistance rates to standard antimicrobial agents has been a very challenging and global health burden. The indiscriminate and widespread antimicrobial use continues to cause significant increase in drug-resistant and multidrug-resistant bacteria [1, 2]. Medicinal plants have long been used in traditional medicine for treatment of various ailments including infectious diseases and many potent phytochemicals or secondary metabolites possessing antimicrobial effects have been isolated from plants [3, 4]. These constituents could serve as veritable lead compounds in the science of drug discovery, development, and research. An example is the startling discovery of penicillin from a microscopic plant in 1928 that lead to the synthesis of its derivatives such as penicillin G [5]. It is quite pertinent to note that since the discovery of nalidixic acid in 1962, which led to the synthesis of more potent fluoroquinolones and derivatives [6], there has not been the introduction of any major pharmacological class of antibacterial agents. Hence, this is posing a great challenge to researchers in the area of drug discovery and development of anti-infective agents and has equally lend credence to the intensified research going on in the area of natural products for the isolation of potent compounds that could serve as lead in the discovery of new antibacterial agents [7]. Screening of medicinal plants and other natural products has led to the isolation of clinically active antibacterial agents [8]. Interestingly, many plant extracts have shown to possess antimicrobial effects and are being used in traditional medicine [9, 10]. *Annona senegalensis* Pers. (Annonaceae) is among the medicinal plants that have been documented to possess antibacterial effects [11–13]. Also the ethnomedicinal uses of the plant in the treatment of wounds and infectious diseases such as diarrhea [14, 15] periodontal and other oral infections [16] had been reported. Furthermore, the anticonvulsant, sedative, and muscle relaxant [17, 18] as well as anti-inflammatory [19] effects of the root bark extract and fractions of *A. senegalensis* have been reported.

Therefore, the objective of this study was to ascertain the antimicrobial effects of the root bark extracts and fractions of *A. senegalensis* and to isolate and characterize the active phytochemical(s) responsible for these effects using proton-NMR and X-ray crystallography.

## 2. Materials and Methods

### 2.1. Plant Materials

Fresh roots of *A. senegalensis* were collected from Enugu-Ezike, Enugu State, Nigeria in the month of June, 2007 and authenticated by a taxonomist, Mr. A. O. Ozioko, of the International Centre for Ethnomedicine and Drug Development (InterCEDD), Aku Road, Nsukka, Enugu State, Nigeria. A voucher specimen was deposited at the InterCEDD herbarium (specimen number: BDCP/INTERCEED–64). 

### 2.2. Test Organisms

Clinical strains of *Escherichia coli, Bacillus subtilis, Pseudomonas aeruginosa, Salmonella paratyphi* and* Staphylococcus aureus*, *Aspergillus*,* niger and Candida albicans,* obtained from the Medical Laboratory Department of Bishop Shanahan Memorial Hospital, Nsukka, Enugu State, Nigeria and preserved in the Microbiology Unit of the Department of Pharmaceutics and Pharmaceutical Microbiology, University of Nigeria, Nsukka, were used. These clinical strains were isolated from designated biological fluids or sources as shown in Table 1.

### 2.3. Animals

Adult albino mice (18–30 g; *n* = 14) bred in the Laboratory Animal Facility of the Department of Pharmacology and Toxicology, University of Nigeria, Nsukka, were used in the studies. The animals were maintained under standard laboratory conditions and had free access to standard pellets (Guinea Feeds, Nigeria Plc) and water. On transfer to the work area, animals were allowed two weeks of acclimatization before the commencement of the experiments. All animal experiments were conducted in compliance with the National Institute of Health Guidelines for Care and Use of Laboratory Animals (Publication no. 85–23, revised 1985) and approval of the University Ethical Committee on the use of laboratory animals.

### 2.4. Preparation and Extraction of Plant Materials

The root barks were peeled off, cut into pieces, and dried under shade. The dried root-bark was then pulverized into coarse powder using a hammer mill. The powdered material (2.95 kg) was extracted with a mixture of methanol: methylene chloride (1 : 1) using Soxhlet extractor to obtain the methanol: methylene chloride extract or the crude extract (MME). This was concentrated at reduced pressure using a rotary evaporator to obtain a yield of 375 g (12.71% w/w).

### 2.5. Solvent-Guided Fractionation of MME and Bioactivity-Guided Studies

The methanol-methylene chloride extract (MME; 250 g) was subjected to solvent-guided fractionation in a silica gel (70-220 mesh, Merck Germany) column, successively eluted with n-hexane, ethyl acetate, and methanol in order of increasing polarity. The fractions were concentrated under reduced pressure in a rotary evaporator (below 40°C) to obtain the hexane fraction (HF; 115 g; 46.0% w/w), ethyl acetate fraction (EF; 61 g; 24.4% w/w), and methanol fraction (MF; 69.5 g; 27.8% w/w). Bioactivity-guided studies on the extract and fractions using agar well diffusion method showed that EF had a potent antibacterial activity with a relatively higher inhibition zone diameter (IZD) than the HF and MF. Subsequently, the ethyl acetate (EF) soluble fraction was subjected to column chromatographic separation. The EF (50 g) was separated in a dry-packed silica gel (70–220 mesh, Merck Germany) column of width 4 cm and length 40 cm. The extract was mixed with the silica gel and loaded on top of the prepacked column. The column was successively eluted with gradient mixtures of n-hexane and ethyl acetate (1 : 0, 9 : 1, 8 : 2, 7 : 3, 6 : 4, 5 : 5, 4 : 6, 3 : 7, and 2 : 8) and the fractions collected in 500 mL volume. The fractions were subsequently pooled and concentrated to afford eight broad fractions, F1–F8, based on the similarity of constituents visualized on silica gel pre-coated TLC plates (Uniplate-Analtech Co., USA), developed with mixtures of n-hexane and ethyl acetate accordingly. Fraction F1 gave an oily liquid or lipophilic fraction, while F2 (9–12; 2000 mL) when concentrated yielded white crystals. However, the crystals yielded by F2 were harvested and purified by repeated washing with n-hexane and dried to obtain *A. senegalensis* crystals (AS2) (2.8 g; 5.6% w/w) which was stored in a refrigerator for activity studies.

### 2.6. Phytochemical Tests

The preliminary phytochemical analysis of methanol-methylene chloride extract (MME), ethyl acetate fraction (EF), hexane fraction (HF), methanol fraction (MF), and ethyl acetate subfraction isolated compound, AS2 were performed using standard phytochemical procedures as described by Harborne [20] and Trease and Evans [21]. Briefly, frothing test for saponins, Salkowski test for terpenoids, Liebermann-Burchard tests for steroids, ferric chloride test for tannins, Keller-Killiani test for cardiac glycosides, Dragendorff's and Mayer's test for alkaloids, Fehling's test for reducing sugars, xanthoproteic test for proteins, iodine test for carbohydrates or starch, and ammonia test for detection of flavonoids were performed for qualitative identification of the phytoconstituents present in [22]. All reagents for the preliminary phytochemical analysis were freshly prepared.

### 2.7. Identification and Characterization of AS2

Structural elucidation of the pure crystals, AS2, was performed using proton NMR and X-ray crystallography, since the compound is in crystal form. The melting point of AS2 was also determined using an analog melting point apparatus (Electrothermal, Cat. no. IA 6304, England). The identity was established by comparison of the spectral data and X-ray crystallography of previously published compounds [23].

### 2.8. Acute Toxicity and Lethality (LD_50_) Test of AS2

The oral acute toxicity and lethality test (LD50) of the AS2 was performed in mice using the method described by Lorke [24]. Briefly, the test was performed in two stages. In stage one, animals received oral administration of one of 10, 100, and 1000 mg/kg (*n* = 3) of AS2 and observed for 24 h for number of deaths. Since no death occurred in any of the groups in the first stage of the test, in stage two of the test, 1600, 2900, and 5000 mg/kg doses of the AS2 were administered to a fresh batch of animals (*n* = 1) and was also monitored for 24 h. Since death occurred at the maximum dose (5000 mg/kg), the LD_50_ was estimated as the product of the square root of the dose that recorded death and the dose that recorded no death preceding it (in this case, 2900 mg/kg dose). We have estimated and reported the acute toxicities and lethality tests of MME, EF, HF, and MF in a separate study [18].

### 2.9. GCMS Analysis of the Lipophilic Fraction (F1)

The gas chromatography mass spectrometry (GCMS) of F1, the lipophilic subfraction, of the EF fractions was analyzed using GCMS-QP2010 PLUS (SHIMADZU, JAPAN) in order to characterize the lipophilic components. Sample of the F1 was suspended in 1 mL of ethyl acetate (Merck, Germany) and 1 : l of this solution was analyzed by the gas chromatography coupled with mass spectrometry equipped with a fused silica capillary column DB-5 (30 m × 0.25 mm × 0.25 m). The electron impact technique (70 eV) was used with the injector temperature at 240°C and that of the detector at 230°C. The carrier gas was helium at the working rate of 1.7 mL/min. The column temperature was initially 60°C and then was gradually increased at the rate of 3 °C/min up to 240°C. For detection of the oil components, we used a flame ionization detector set up at 230°C. The identification of the components of the lipophilic fraction was effected through comparison of substance mass spectrum with the database of the GC/MS (NIST 62.lib), the literature, and retention index [25, 26].

### 2.10. Antimicrobial Assay

Each of the extract (MME), fractions (HF, EF, and MF), F1 and AS2 was dissolved in dimethyl sulfoxide (DMSO) to obtain 100 mg/mL concentration. Subsequently, the concentration was diluted to obtain 50, 25, 12.5, and 6.25 mg/mL for the determination of the minimum inhibitory concentration (MIC) at the dose levels. Agar well diffusion method as described in [27, 28] was employed for the assay. The test organisms, the clinical isolates, were prepared with a 0.5 McFarland standard and subcultured at 37°C and maintained on nutrient agar media for bacteria and sabouraud agar media for fungi (*Aspergillus niger* and *Candida albicans*). Petriplates containing 20 mL of respective medium were seeded with selected microbial strains and incubated at 37°C for 24 hours. Standard antimicrobial agents used as positive controls were gentamycin (Lek, Slovakia) and ciprofloxacin (Medreich, India). After 24 hours the inhibition zone diameters (IZD) were recorded and the mean calculated. The minimum inhibitory concentrations (MICs) were then determined at various dilutions by extrapolation from the graphs of IZD squared (IZD^2^) against logarithm of the concentration. 

### 2.11. Statistical Analysis

Data obtained were analysed by SPSS (Version 14) using One-Way Analysis of Variance (ANOVA) with Dunnet its test for multiple comparisons with the control. Values are in mean ± SEM and were considered significant at *P* < 0.05. 

## 3. Results

### 3.1. Phytochemical Tests

Phytochemical tests of methanol-methylene chloride extract (MME) gave positive reactions with phytochemical reagents with respect to alkaloids, carbohydrates, flavonoids, fats and oils, glycosides, reducing sugars, resins, steroids, saponins, and terpenoids. The ethyl acetate (EF) gave positive reactions for alkaloids, flavonoids, resins and terpenoids, while AS2 gave a strong positive reaction with Salkowski test. The phytoconstituents of hexane fraction (HF) and methanol fraction (MF) were also shown (Table 2).

### 3.2. Acute Toxicity and Lethality (LD_50_) Test of AS2

The medium lethal dose (LD_50_) of the AS2 was found to be 3800 mg/kg in mice indicating the good level of safety.

### 3.3. Identification and Characterization of AS2

**Figure 1:**
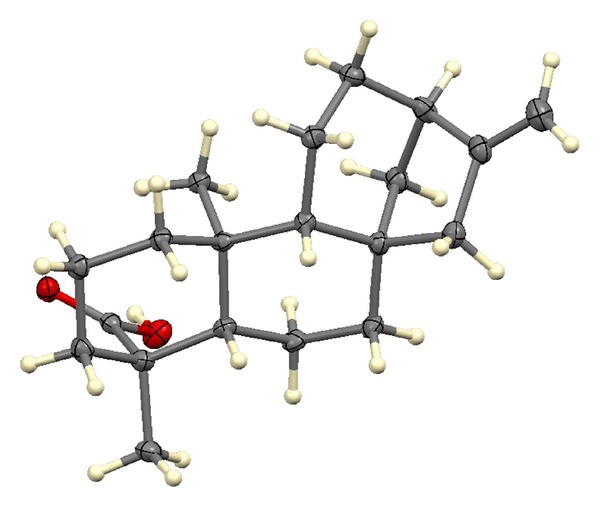
X-ray crystallograph of AS2.

**Figure 2:**
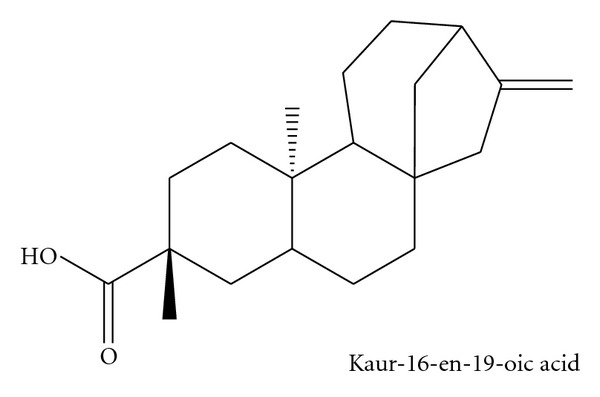
Chemical structure of AS2.

**Figure 3:**
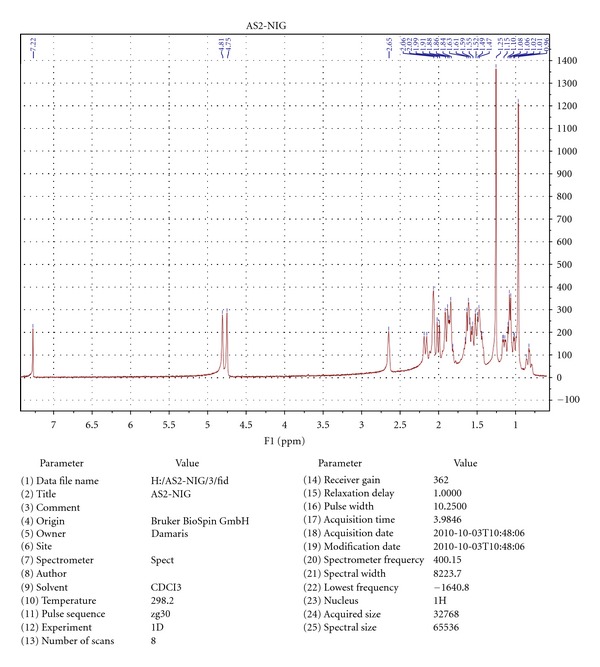
Spectral data analysis of AS2.

The AS2 was shown to be a white crystalline and odourless compound. The results of the proton NMR and X-ray crystallography, when compared with the spectral data of known compounds, established the identity of AS2 to be kaur-16-en-19-oic acid or kaurenoic acid, a diterpenoid, with the chemical structure and X-ray crystallograph as shown (Figures 1, 2 and 3). The melting point of AS2 was found to be 170–172°C.

### 3.4. GCMS Analysis of the Lipophilic Fraction (F1)

**Figure 4:**
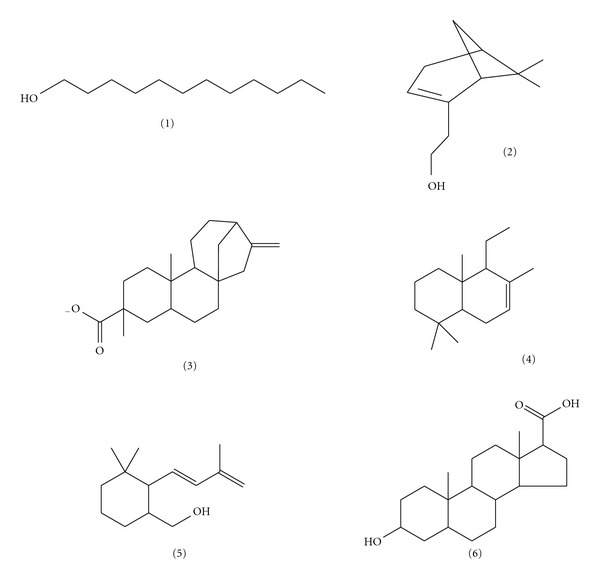
Chemical structures of GCMS constituents of F1.

The GCMS analysis of F1 revealed the presence of the following 6 major constituents which include kaur-16-en-19-oic acid, 1-dodecanol, 1-naphthalenemethanol, 6,6-dimethyl-bicyclo [3.1.1]hept-2-ene-2-ethanol, 3,3-dimethyl-2-(3-methylbuta-1,3-dienyl)cyclohexan-1-methanol, and 3-hydroxyandrostan-17-carboxylic acid (Table 3). The chemical structures of these constituents are shown in Figure 4.

### 3.5. Antimicrobial Assay

The extract, fractions, and AS2 exhibited significant antibacterial activity and are devoid of any antifungal activity. The results of the inhibition zone diameter (IZD) and MICs of the extract and fractions revealed activity against gram-positive and gram-negative organisms (Table 4). The order of potency against the various bacteria isolates with respect to their MICs by the extract and fractions was *B. subtilis* (AS2 > F1 > HF *≡* MF > EF > MME), *S. aureus* (AS2 > MME), and *P. aeruginosa* (F1 > MME) (Table 5). The MME exhibited an MIC of 0.370, 8.75, 1.08, and 0.07 mg/mL against the clinical isolates of *B. subtilis, S. aureus*, *P. aeruginosa*, and *S. typhi*, respectively. The extracts and isolate showed no antibacterial activity against *E. coli* (Table 4). The F1 gave an MIC of 0.06 and 0.04 mg/mL against *B. subtilis* and *P. aeruginosa*, respectively. AS2 has potency against *S. aureus* and *B. subtilis* with an MIC of 0.15 and 0.03 mg/mL, respectively, whereas EF offered an MIC of 0.18 mg/mL against *B. subtilis* (Table 5). However, the antifungal test for MME, EF, HF, MF, F1, and AS2 against *A. niger* and *C. albicans* showed no activity.

## 4. Discussion

The antimicrobial effects of the methanol-methylene chloride extract (MME), ethyl acetate fraction (EF), hexane fraction (HF), methanol fraction (MF), the lipophilic sub-fraction (F1), and the isolated compound, AS2 exhibited appreciable antibacterial effects but are devoid of antifungal activity. However, AS2 exhibited the lowest MIC value of 30 *μ*g/mL against *B. subtilis* and therefore exhibited the most potent activity when compared to the extracts and fraction of the root bark of *A. senegalensis*. The results of the proton NMR and X-ray crystallography identified and characterized AS2 to be a diterpene known as kaur-16-en-19-oic acid or kaurenoic acid (KA). The melting point of AS2 was found to be 170–172°C which was of a comparable range with that of kaurenoic acid from a different source already reported [29]. In a separate study, the antibacterial effects of the essential oil from *A. senegalensis* have been reported [12] and more importantly the antibacterial activity of kaurenoic acid from the root extract of another plant, *Viguiera arenaria,* has been documented [30]. The extracts, F1 and the KA, exhibited better antibacterial effects against gram-positive organisms such as *B. subtilis* and *S. aureus*, than the gram-negative rods such as *P. aeruginosa*, *S. paratyphi*, and *E. coli* used in the study.

Therefore, the antibacterial effects of MME, EF, HF, MF, F1, and AS2 against organisms such as *P. aeruginosa* and *S. aureus* correlated with the ethnomedicinal use of the plant in wound healing, since *P. aeruginosa* and *S. aureus* had been implicated in the contamination of wounds and boils [31]. The antibacterial activity against *P. aeruginosa* is of interest because *P. aeruginosa* has been reported to be resistant to many antibacterial agents and identified as an opportunistic pathogen which causes complications in immune-compromised patients [32]. The activity of root bark extracts of *A*. senegalensis against *S. aureus* has also been documented in a separate study [13]. Moreover, according to Apak and Olila [13], the root bark extract of *A. senegalensis* exhibited no activity against *E. coli* which was consistent with the lack of activity of MME, EF, HF, MF, F1, and AS2 against *E. coli* identified in this study.

In addition to the antibacterial action of KA, there is the possibility of the presence of other phytochemicals contributing to the antibacterial activity of the extracts and fractions of *A. senegalensis*. This is more so since the F1, which consisted 6 major constituents, together with KA, exhibited potent antibacterial activity with an MIC of 40 *μ*g/mL against *P. aeruginosa*, whereas the kaurenoic acid alone did not show appreciable activity against *P. aeruginosa*. Hence, due to the presence of six major phytochemicals in the GC-MS analysis of the F1, the observed antibacterial effects could be attributed to the combined or single effects of any of the compounds which include kaur-16-en-19-oic acid, 1-dodecanol, 1-naphthalenemethanol, 6,6-dimethyl-bicyclo [3.1.1] hept-2-ene-2-ethanol, 3,3-dimethyl-2-(3-methylbuta-1,3-ienyl)cyclohexan-1-methanol, and 3-hydroxyandrostan-17-carboxylic acid. The presence of kaurenoic acid among these compounds is of practical interest as it was also established as the pure isolated compound, AS2. The kaurenoic acid from the F1 gave the following mass ions on fragmentation with the GCMS analysis: 316 (molecular ion), 301 (demethylation), and 273 (decarboxylation). Notably, steroids, organic acids, and alcohols dominated the remaining 5 compounds of the F1 and both organic acids as well as alcohols are known to possess antibacterial activity especially at higher concentrations [3]. However, F1 showed a strong indication and possibility of being a fixed oil, since it possesses some characteristics attributable to fixed oils which include odorless liquid at room temperature, pale yellowish in colour, permanent grease spot on filter paper when heated in an oven and often contains mixture of organic acids as well as being an extraction product of n-hexane or mixture of n-hexane and ethyl acetate [33]. Medicinal plants with preponderance of variety of secondary metabolites, such as tannins, terpenoids, essential oils, alkaloids, and flavonoids have been found *in vitro*, to possess antimicrobial properties [3]. There is a recognizable loss of activity due to fractionation as the crude extract, MME, exhibited activity against all the organisms tested except *E. coli*, whereas the fractions were found to have lost activity to some of the tested organisms especially *S. paratyphi*. The presence of diterpenoid compounds in the extracts and fractions of *A. senegalensis* really correlated well with the high presence of resins in plants as diterpene acids occur well in plant resins. Some other published works have reported the isolation of kaurenoic acid from the leaves of *A. senegalensis* [34, 35] and aerial parts of *Espeletia semigloburata* [36] which exhibited antibacterial, anticancer, anti-inflammatory, and antipyretic effects. The lack of activity against fungal organism, *C. albicans*, in the study was in consistent with other reported work on root bark extract of *A. senegalensis* which exhibited lack of activity against *C. albicans* [16]. In other documented studies, terpenoids have shown to possess antibacterial [3, 37, 38], antiviral [39, 40], and antiprotozoal effects [41]. Particularly, antibacterial effects of diterpenoids isolated from other plants have been reported [42, 43]. The results from this study tend to support the ethnomedicinal claim of root bark of *A. senegalensis* in treatment of bacterial infections and wound healing particularly in the treatment urinary tract infections in veterinary animals [13]. Furthermore, KA could equally serve as a veritable lead compound in the development of potent standard antibacterial agent.

## 5. Conclusion

Results of the study have indicated that a diterpenoid, kaur-16-en-19-oic acid or kaurenoic acid, has been identified as the phytochemical constituent responsible for the antibacterial effects of root bark of Nigerian *Annona senegalensis* Pers. (Verbenaceae) and is devoid of appreciable antifungal effects.

## Figures and Tables

**Table 1:** Biological sources of clinical strains of the test organisms.

S/No	Clinical strain	Biological source
1	*Bacillus subtilis*	Wound
2	*Escherichia coli*	Stool
3	*Pseudomonas aeruginosa*	Wound
4	*Salmonella paratyphi*	Stool
5	*Staphylococcus aureus*	Nasal discharge
6	*Aspergillus niger*	Spoiled food
7	*Candida albicans*	High vaginal swab (HVS)

**Table 2:** Phytochemical constituents of extract and fractions.

Constituent	MME	EF	HF	MF	AS2
Carbohydrate	+	−	−	+	−
Alkaloid	+	+	+	+	−
Reducing sugar	+	−	−	+	−
Glycoside	+	−	−	+	−
Saponins	+	−	−	+	−
Tannins	−	−	−	−	−
Flavonoids	+	+	−	−	−
Resin	+	+	+	+	−
Fats and oils	+	+	+	−	−
Steroids	+	−	+	+	−
Terpenoids	+	+	+	+	+
Acidic compounds	+	+	−	−	−

^+^ = present; ^−^ = absent.

**Table 3:** Chemical constituents of lipophilic fraction (F1).

S/No	Chemical name	Molecular formula	Molecular weight	Retention (KOVAT's) index	MS ions/fragments (m/e)
01	1-dodecanol	C_12_H_26_O	186	1457	27, 41, 55, 70, 83, 97, 111(112), 126, 140
02	6,6-dimethyl-bicyclo [3.1.1]hept-2-ene-2-ethanol	C_11_H_18_O	166	1290	27, 41 (43), 67, 79, 91, 105, 122, 151.
03	Kaur-16-en-18-oic acid	C_21_H_32_O_2_	316	2056	27, 41, 55, 67, 79, 91, 105, 121, 133, 147, 159, 187, 213, 241, 257, 273, 301, 316.
04	1-naphthalenemethanol	C_15_H_26_O	222	1685	27, 41, 55, 69, 81, 109, 124, 191, 222.
05	3,3-dimethyl-2-(3-methylbuta-1,3-dienyl) cyclohexan-1-methanol	C_14_H_24_O	208	1592	41, 55, 69, 81, 95, 109, 123, 139, 165, 177, 193.
06	3-hydroxyandrostan-17-carboxylic acid	C_20_H_32_O_3_	320	2375	43, 67, 79, 93, 108, 121, 135, 147, 161, 175, 194, 215, 233, 248, 287, 302.

**Table 4:** Inhibition zone diameter (IZD) of the extract and fractions.

Organism	MME	HF	MF	EF	F1	AS2	CIP	GEN
*B. subtilis *	14.60 ± 0.12	13.30 ± 0.15	13.30 ± 0.06	14.00 ± 0.00	14.00 ± 0.23	16.00 ± 0.00	22.00 ± 0.12	26.00 ± 0.00
*S. aureus *	14.20 ± 0.42	12.30 ± 0.35	+	10.50 ± 0.17	10.33 ± 0.06	17.00 ± 0.00	28.00 ± 0.00	26.00 ± 0.00
*P. aeruginosa*	13.00 ± 0.00	+	+	+	11.40 ± 0.31	+	25.00 ± 0.00	22.00 ± 0.12
*S. paratyphi *	12.60 ± 0.20	+	+	+	+	+	25.00 ± 0.30	20.00 ± 0.00
*E. coli*	+	+	+	+	+	+	NA	NA
*A. niger*	+	+	+	+	+	+	NA	NA
*C. albicans*	+	+	+	+	+	+	NA	NA

Values in mm, are mean ± SEM (ANOVA, Dunnet its *post hoc*); *n* = 3; ^+^: no growth or no activity; concentration of AS2 = 50 mg/mL; CIP & GEN: 40 *μ*g/mL while others are 100 mg/mL. CIP: ciprofloxacin, GEN: gentamicin, NA = not applicable.

**Table 5:** Minimum inhibitory concentration (MIC) of extract and fractions.

ORGANISM	MME	MF	HF	EF	F1	AS2	CIP	GEN
*B. subtilis*	370.00	150.00	140.00	180.00	60.00	30.00	0.28	0.02
*S. aereus*	8750.00	+	+	+	+	150.00	1.18	0.23
*P. aeruginosa*	1080.00	+	+	+	40.00	+	3.60	0.79
*S. paratyphi*	70.00	+	+	+	+	+	0.39	1.77

MIC values are in *μ*g/mL.

## References

[1] Livermore D (2004). Can better prescribing turn the tide of resistance?. *Nature Reviews Microbiology*.

[2] Choi JG, Jeong SI, Ku CS (2009). Antibacterial activity of hydroxyalkenyl salicylic acids from sarcotesta of ginkgo biloba against vancomycin-resistant enterococcus. *Fitoterapia*.

[3] Cowan MM (1999). Plant products as antimicrobial agents. *Clinical Microbiology Reviews*.

[4] Salazar-Aranda R, Perez-Lopez L, Lopez-Arroyo J, Waksman de Torres N (2011). Antimicrobial and antioxidant activities of plants from Northeast of Mexico. *Evidence-Based Complementary and Alternative Medicine*.

[5] Sofowora AS (2008). *Medicinal Plants and Traditional Medicine in Africa*.

[6] Lescher GY, Froelich ED, Gruet MD, Bailey JH, Brundage RP (1962). 1,8-Naphthyridine derivatives: a new class of chemotherapeutic agents. *Journal of Medical and Pharmaceutical Chemistry*.

[7] Yuvaraj G, Sathyanathan V, Shankar S, Kumar NR (2010). Anticancer and *in-vitro* activities of Derris brevipes Var brevipes. *Journal of Chemical and Pharmaceutical Research*.

[8] Bibi Y, Nisa S, Chaudhary FM, Zia M (2011). Antibacterial activity of some selected medicinal plants of pakistan. *BMC Complementary and Alternative Medicine*.

[9] Brantner A, Grein E (1994). Antibacterial activity of plant extracts used externally in traditional medicine. *Journal of Ethnopharmacology*.

[10] Assob JCN, Kamga HLF, Nsagha DS (2011). Antimicrobial and toxicological activities of five medicinal plant species from Cameroun traditional medicine. *BMC Complementary and Alternative Medicine*.

[11] Muanza DN, Kim BW, Euler KL, Williams L (1994). Antibacterial and antifungal activities of nine medicinal plants from zaire. *International Journal of Pharmacognosy*.

[12] Samie A, Obi CL, Bessong PO, Namrita L (2005). Activity profiles of fourteen selected medicinal plants from rural venda communities in south africa against fifteen clinical bacterial species. *African Journal of Biotechnology*.

[13] Apak L, Olila D (2006). The *in-vitro* antibacterial activity of *Annona senegalensis*, *Securidacca longipendiculata* and *Steganotaenia araliacea*—Ugandan medicinal plants. *African Health Sciences*.

[14] Kudi AC, Myint SH (1999). Antiviral activity of some Nigerian medicinal plant extracts. *Journal of Ethnopharmacology*.

[15] Suleiman MM, Dzenda T, Sani CA (2008). Antidiarrhoeal activity of the methanol stem-bark extract of *Annona senegalensis* pers. (Annonaceae). *Journal of Ethnopharmacology*.

[16] More G, Tshikalange TE, Lall N, Botha F, Meyer JJM (2008). Antimicrobial activity of medicinal plants against oral microorganisms. *Journal of Ethnopharmacology*.

[17] Okoye TC, Akah PA, Omeke CP (2010). Evaluation of the anticonvulsant and muscle relaxant effects of the methanol root bark extracts of *Annona senegalensis*. *Asian Pacific Journal of Tropical Medicine*.

[18] Okoye TC, Akah PA (2010). Anticonvulsant and sedative effects of root bark extract and fractions of *Annona senegalensis*. *Inventi Impact*.

[19] Okoye TC, Akah PA, Ezike AC, Nwoye JC (2011). Studies on the effects of *Annona senegalensis* root bark extract on acute and chronic inflammation in rats. *Journal of Pharmacy Research*.

[20] Harborne JB (1988). *Phytochemical Methods: A Guide to Modern Techniques of Plant Analysis*.

[21] Trease GE, Evans WC (1989). *Test book of Pharmacognosy*.

[22] Sofowora AS (2008). *Medicinal Plants and Traditional Medicine in Africa*.

[23] Brassy C, Bachet B, Wollenweber E (1988). Acide decahydro-1,2,3,4,5,6,7,8,9,10-dimethyl-1,4a (methylene-1) ethano-7,8a phenanthrenecarboxylique-1, acide (-)- kaurene-16 oique-19. *Acta Crystallographica C*.

[24] Lorke D (1983). A new approach to practical acute toxicity testing. *Archives of Toxicology*.

[25] McLafferty FW, Stauffer D (1989). *The Wiley/NBS Registry of Mass Spectral Data, vol. 1-2*.

[26] Adams RP (1995). *Identification of Essential Oil Components by Gas Chromatography/ Mass Spectroscopy*.

[27] Lovian V (1980). *Antibiotics in Laboratory Medicine*.

[28] Perez C, Paul M, Bazerque P (1990). An antibiotic assay by the agar well-diffusion method. *Acta Biologiae et Medicine Experimentalis*.

[29] Daló NL, Sosa-Sequera MC, Usubillaga A (2007). On the anticonvulsant activity of kaurenic acid. *Investigacion Clinica*.

[30] Porto TS, Rangel R, Furtado NAJC (2009). Pimarane-type diterpenes: antimicrobial activity against oral pathogens. *Molecules*.

[31] Okoli CO, Akah PA, Okoli AS (2007). Potentials of leaves of *Aspilia africana* (Compositae) in wound care: an experimental evaluation. *BMC Complementary and Alternative Medicine*.

[32] Adeshina GO, Tahir TS, Onaolapo JA (2010). Microbiological evaluation of packaged pineapple juice marketed in Kaduna metropolis. *Nigerian Journal of Pharmaceutical Research*.

[33] Mukherjee PK (2007). *Quality Control of Herbal Drugs: An Approach to Evaluation of Botanicals*.

[34] Eshiet ITU, Akisanya A, Taylor DAH (1971). Diterpenes from *Annona senegalensis*. *Phytochemistry*.

[35] Fatope MO, Audu OT, Takeda Y (1996). Bioactive ent-kaurene diterpenoids from *Annona senegalensis*. *Journal of Natural Products*.

[36] Sosa-Sequera MC, Suárez O, Daló NL (2010). Kaurenic acid: an *in vivo* experimental study of its anti-inflammatory and antipyretic effects. *Indian Journal of Pharmacology*.

[37] Kubo I, Muroi H, Himejima M (1992). Antibacterial activity of totarol and its potentiation. *Journal of Natural Products*.

[38] Mendoza L, Wilkens M, Urzúa A (1997). Antimicrobial study of the resinous exudates and of diterpenoids and flavonoids isolated from some chilean *Pseudognaphalium* (Asteraceae). *Journal of Ethnopharmacology*.

[39] Pengsuparp T, Cai L, Fong HHS (1994). Pentacyclic triterpenes derived from *Maprounea africana* are potent inhibitors of hiv-1 reverse transcriptase. *Journal of Natural Products*.

[40] Sun HD, Qiu SX, Lin LZ (1996). Nigranoic acid, a triterpenoid from *Schisandra sphaerandra* that inhibits hiv-1 reverse transcriptase. *Journal of Natural Products*.

[41] Ghoshal S, Krishna Prasad BN, Lakshmi V (1996). Antiamoebic activity of piper longum fruits against *Entamoeba histolytica* in vitro and in vivo. *Journal of Ethnopharmacology*.

[42] Batista O, Duarte A, Nascimento J, Simoes MF (1994). Structure and antimicrobial activity of diterpenes from the roots of *Plectranthus hereroensis*. *Journal of Natural Products*.

[43] Kadota S, Basnet P, Ishii E, Tamura T, Namba T (1997). Antibacterial activity of trichorabdal a from *Rabdosia trichocarpa* against *Helicobacter pylori*. *Zentralblatt Fur Bakteriologie*.

